# Immune subtype identification and multi-layer perceptron classifier construction for breast cancer

**DOI:** 10.3389/fonc.2022.943874

**Published:** 2022-12-08

**Authors:** Xinbo Yang, Yuanjie Zheng, Xianrong Xing, Xiaodan Sui, Weikuan Jia, Huali Pan

**Affiliations:** ^1^ School of Information Science and Engineering, Shandong Normal University, Jinan, China; ^2^ Department of Pharmacy, Shandong Medical College, Jinan, China; ^3^ Business School, Shandong Normal University, Jinan, China

**Keywords:** breast cancer, immune infiltration, subtype identification, tumor mutational burden, multi-layer perceptron classifier

## Abstract

**Introduction:**

Breast cancer is a heterogeneous tumor. Tumor microenvironment (TME) has an important effect on the proliferation, metastasis, treatment, and prognosis of breast cancer.

**Methods:**

In this study, we calculated the relative proportion of tumor infiltrating immune cells (TIICs) in the breast cancer TME, and used the consensus clustering algorithm to cluster the breast cancer subtypes. We also developed a multi-layer perceptron (MLP) classifier based on a deep learning framework to detect breast cancer subtypes, which 70% of the breast cancer research cohort was used for the model training and 30% for validation.

**Results:**

By performing the K-means clustering algorithm, the research cohort was clustered into two subtypes. The Kaplan-Meier survival estimate analysis showed significant differences in the overall survival (OS) between the two identified subtypes. Estimating the difference in the relative proportion of TIICs showed that the two subtypes had significant differences in multiple immune cells, such as CD8, CD4, and regulatory T cells. Further, the expression level of immune checkpoint molecules (PDL1, CTLA4, LAG3, TIGIT, CD27, IDO1, ICOS) and tumor mutational burden (TMB) also showed significant differences between the two subtypes, indicating the clinical value of the two subtypes. Finally, we identified a 38-gene signature and developed a multilayer perceptron (MLP) classifier that combined multi-gene signature to identify breast cancer subtypes. The results showed that the classifier had an accuracy rate of 93.56% and can be robustly used for the breast cancer subtype diagnosis.

**Conclusion:**

Identification of breast cancer subtypes based on the immune signature in the tumor microenvironment can assist clinicians to effectively and accurately assess the progression of breast cancer and formulate different treatment strategies for different subtypes.

## 1 Introduction

Breast cancer is a disease with high morbidity and mortality, only lower than lung cancer in women (1, 2). According to a report by the American Cancer Society in 2019, in the last 5 years (2012-2016), the incidence of breast cancer has increased slightly at a rate of 0.3% per year (3). Breast cancer is a highly heterogeneous tumor (4); the tumor tissue not only includes tumor cells, but also normal epithelial, stromal, and immune cells that are associated with tumors. The tumor microenvironment (TME) that is composed of these cells has an important impact on the tumor proliferation, metastasis, treatment, and prognosis (5–7).

Immune cells are scattered in the tumor center and infiltration margin or adjacent tertiary lymphoid tissues, and can be roughly divided into immunosuppressive and immune effector cells (8, 9). The level of immune cells infiltration reflectsthe degree of tumor development, affecting cancer progression (10). The tumor immune microenvironment (TIME) is composed of various cells that can inhibit the tumor formation (11–13) and promote tumorigenesis (14, 15).

Quantification of the proportion of various cells in the TME is important to understand the occurrence and development of tumors. Yoshihara K et al. proposed a method (ESTIMATE) of using gene expression profiles to calculate the ratio of stromal to immunecells to reveal the tumor purity (16). Newman et al. utilized the gene expression data to estimate the abundance of immune cells in tumor samples, and developed the analysis tool CIBERSORT for estimating and verifying the proportion of 22 immune cells (17).

Breast cancer is very difficult to cure; however, early diagnosis and timely treatment can prolong the patients’ survival. Immunotherapy is considered the most promising treatment for breast cancer currently and includes immune checkpoint blocking (ICB) therapy (18, 19), adoptive T cell immunotherapy (20), and tumor vaccine immunotherapy (21, 22). The US FDA has also approved few immunotherapy drugs mainly Keytruda (Pembrolizumab), Opdivo (Nivolumab), Tecentriq (Atezolizumab) among others.

Immunotherapy is not suitable for all breast cancer patients (23–26), and hence, it is important to accurately determine the cancer subtype in such patients so that appropriate treatment can be administered. Perou et al. distinguished the breast cancer subtypes based on the differences in mRNA expression patterns, and proposed, for the first time, that breast cancer can be divided into four subtypes (27). Subsequently, a 50-gene breast cancer classification model (PAM50) was developed based on the gene expression profile data (28), and is commonly employed in clinical practice. Further, based on the molecular subtype identification of triple-negative breast cancer, six (29), four (30), and three (31) subtypes have been proposed, while a model of six subtypes was also proposed based on the colon cancer classification method (32). Although these subtype classification methods elucidated the molecular markers, prognostic differences, and clinical significance of the subtypes; TME and the influence of TIME on the occurrence, development, and prognosis of tumors have not been evaluated. Additionally, the association of immune checkpoint molecules and breast cancer isnot comprehensively understood.

In this study, the ESTIMATE algorithm was used to determine the individual and combined scores of the immune and stromal cells of each sample in the breast cancer research cohort. Further, we used the CIBERSORT algorithm to estimate the scores of the 22 types of immune cells in the same research cohort. We propose a method to identify breast cancer subtypes by combining the estimated scores of the two immune infiltrations. Two breast cancer subtypes were identified using the consensus clustering algorithm, and the survival, immune cell differential, immune checkpoint molecules differential, tumor mutation burden correlation, differential gene enrichment, and drug sensitivity analyses were performed for these two subtypes. We showed that this classification into two subtypes has a potential for clinical application. We also developed a multi-layer perceptron (MLP) classifier based on a deep learning framework to detect two breast cancer subtypes. By using the training data to train the classifier model, the test results showed that the classifier can distinguish the two subtypes.

## 2 Materials and methods

### 2.1 Data search strategy and collection

The breast cancer data used in this study were obtained from the two public databases, The Cancer Genome Atlas (TCGA, https://portal.gdc.cancer.gov/) and Gene Expression Omnibus (GEO, https://www.ncbi.nlm.nih.gov/geo/). The TCGA data included the transcriptome mRNA expression profile data of female patients (n=1208, 1096 cancer and 112 normal samples), clinical data (n=1085), and simple nucleotide variation (SNV) data. The research cohorts selected from the GEO database were GSE42568 (n=104) and GSE88770 (n=117), including the mRNA expression profile files of the patient cohort and probe files of the sequencing platform.

### 2.2 Data preprocessing

TCGA mRNA expression and clinical data were normalized through the following steps: (1) mapping of the mRNA expression data to the human genome annotation file, replacing Ensemble IDs with gene names, and deleting the genes lacking a corresponding mapping, (2) standardization of the mRNA expression data, (3) conversion of FPKM standardization data into TPM standardization; when the same sample was repeated, the average value of gene expression was used instead. Further, the normal samples were deleted, and (4) using perl language scripts to extract the clinical information, including the sample id, overall survival (OS) in days, survival status, age, grade, and stage (T, M, and N staging). The breast cancer data of the GEO database were annotated with theprobe data of the sequencing platform GPL570. We extracted the gene expression data and clinical information separately. Finally, we consolidated and combined the TCGA and GEO expression data.

### 2.3 Estimate the proportion of tumor-infiltrating immune cells and tumor purity

The proportion of 22 TIICs types were estimated by using the CIBERSORT algorithm for each sample, and samples with a p-value of <0.05 were selected for the survival analysis. For tumor purity, we used the ESTIMATE algorithm for evaluation. Two non-tumor components (immune and stromal cells) was calculated by using the ESTIMATE algorithm and gene expression profiles, and obtained three tumor purity signatures (stromal, immune, and estimate scores).

### 2.4 Identification of breast cancer subtypes

The R language “ConsensusClusterPlus” package was used to perform the consistent clustering, and to separately save the graphs of the clustering results for each K value (Integer K, 2≤*K*≤9 ). The parameters of the unsupervised clustering weredefined, including the clustering algorithm (clusterAlg=“km”), maximum number of clusters (maxK=9), number of resampling (reps=50), sampling ratio (pItem=0.8), characteristics sampling ratio (pFeature=1), and clustering distance (distance=“euclidean”).

### 2.5 Statistical analysis

The statistical analysis was performed by Rstudio software, R version 4.1.2. For clinical data, the R packages ßurvival” and ßurvminer” were used for the survival analysis, and the Kaplan-Meier survival curve was drawn. Using the “limma” package, differentially expressed genes (DEGs) between subtypes, as well as the expression differences of immune checkpoint molecules, tumor mutational burden (TMB), and drug sensitivity were statistically analyzed. “ggplot2” was used to draw the graphics and figures.

### 2.6 Breast cancer subtype classifier based on the neural network

Deep learning algorithms are gradually being widely used in the field of biomedicine (33–35). We designed an MLP classifier to identify the breast cancer subtypes. This classifier included an input, hidden, and output layer. The input layer contains 38 nodes, which represent 38 DEGs. The activation function of the multilayer perceptron model uses the “sigmoid”, and the mathematical formula is expressed as:


(1) $$sigmoid=\frac{1}{1+e^{-x}}$$

The loss function of the model used the cross entropy loss function, and the mathematical formula is expressed as:


(2) $$Loss=-\frac{1}{n}\sum_{x}\left[ylna+\left(1-y\right)ln\left(1-a\right)\right]$$

where, *x* represents the sample, *y* represents the true label, *a* represents the predicted output value, and *n* represents the total number of samples.

The optimization of the model uses the RMSProp optimization algorithm and the mathematical formula is expressed as:


(3) $$\begin{array}{c} S_{dw}=\beta S_{dw}+\left(1-\beta \right)dw^{2}\\ w=w-\alpha \frac{dw}{\sqrt{S_{dw}}} \end{array}$$

Where *dw* is the gradient, *S*_*dw*_ is a value container, which stores the result of the square weighted average of all the gradients, *α* is the learning rate (general value: 0.001), *β* decay factor (general value: 0.9). In order to obtain a classifier model with robust performance and high accuracy, we also verified the impact of the number of hidden layer nodes on the classification results, ranging from 2 to 38.

## 3 Results

### 3.1 Estimate of the proportion of TIICs in the breast cancer research cohort

**Figure 1:**
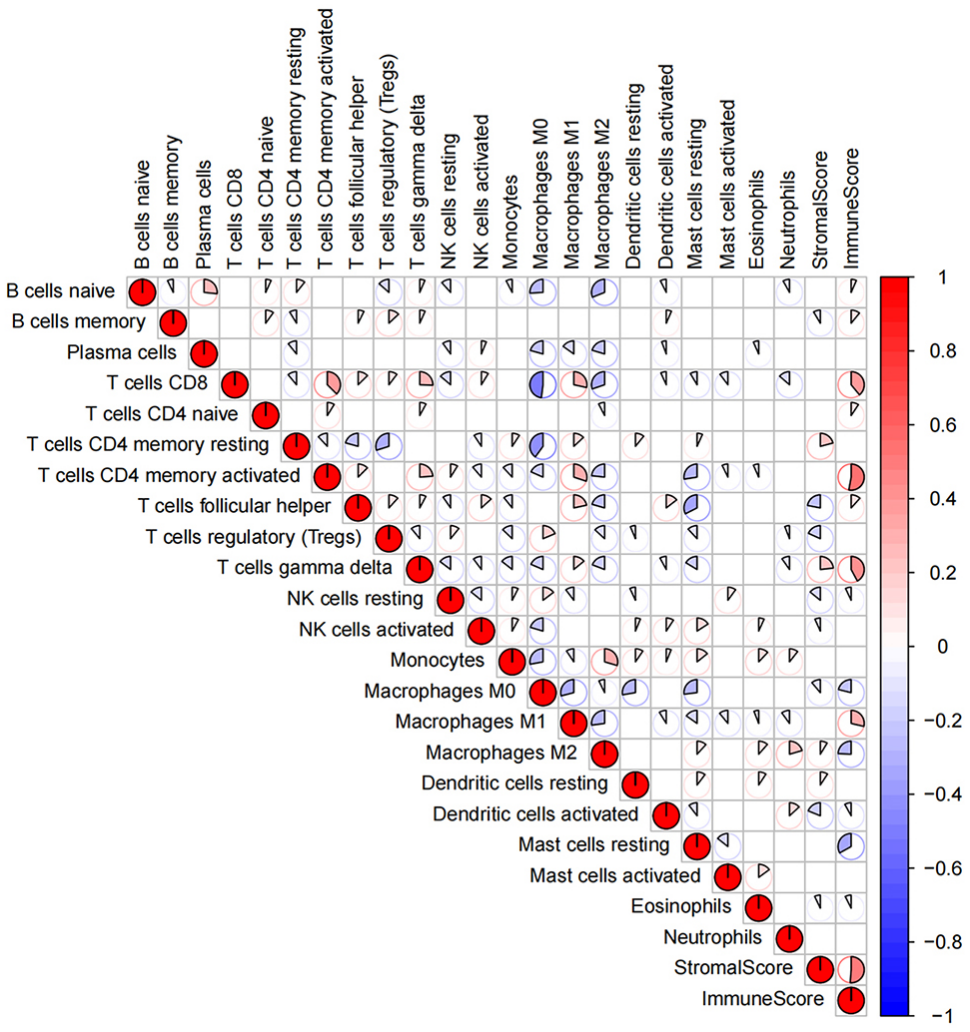
The general landscape of infiltrating immune cells interactions in breast cancer. The color value and shape size of the pie chart represent the correlation size between immune cells. The color bar shows the positive and negative values of the correlation.

The proportion of immune cells and tumor purity in the research cohort were quantified by using CIBERSORT and ESTIMATE algorithms (**Table S1**); based on the quantitative score, the general landscape of TIICs interaction in breast cancer TME was visualized by generating the correlation coefficient heat map (**Figure 1**). The correlation analysis of TIICs showed that the CD8 and memory-activated CD4 T cells and M0 macrophages had the strongest positive and negative correlations, respectively. In addition, M1 macrophages and CD8, memory-activated CD4, and follicular helper T Cells showed strong positive correlations.

### 3.2 Subtype clustering and differential analysis of immune cells

**Figure 2:**
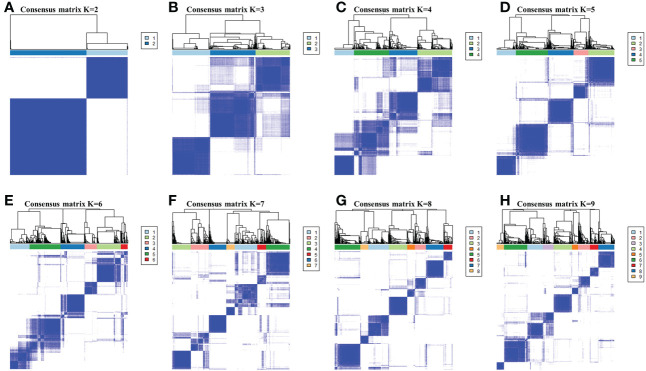
**(A-H)** respectively showed the consensus matrices of all breast cancer samples in the research cohort for each k (2≤*k*≤9 ), displaying the clustering stability by performing 100 iterations of hierarchical clustering. Perform subtype clustering using the K-means clustering algorithm, with k in the range of 2 to 9.

**Figure 3:**
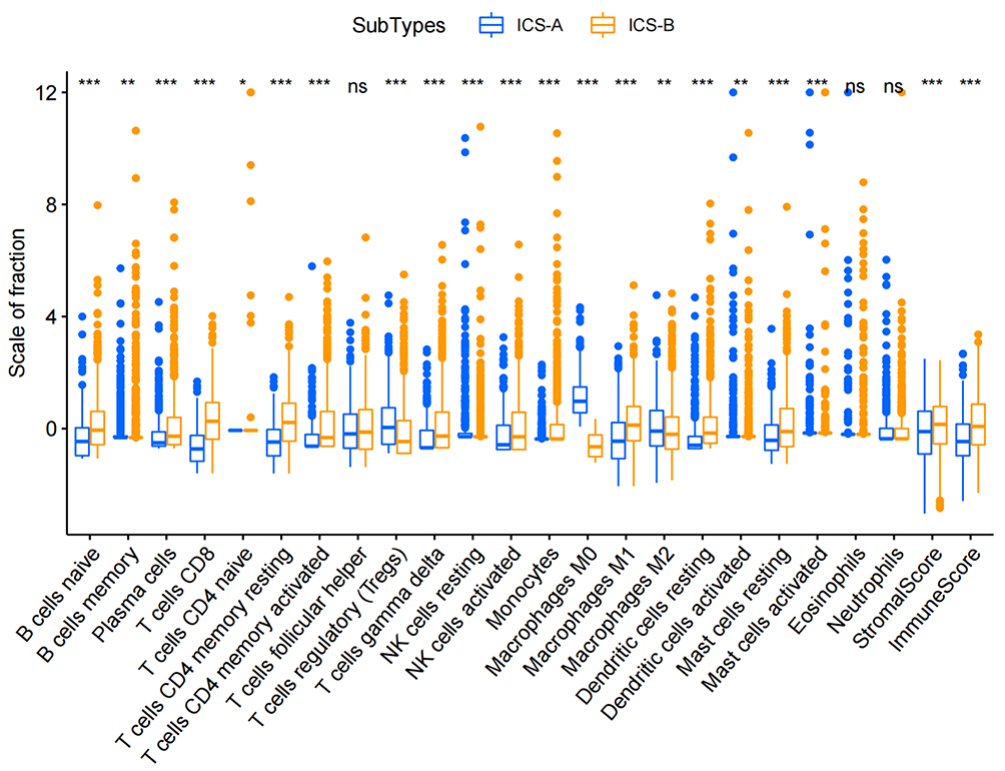
Boxplots showing statistical differences in immune cells in the two immune subtypes ICS-A and ICS-B. Comparison was performed using the Wilcoxon signed-rank test. *p < 0.05, **p < 0.01, ***p < 0.001 (p-values were adjusted using FDR correction).

By performing the K-means clustering algorithm, 8 cluster maps were generated (**Figures 2A–H**). **Figure 2A** shows two subtypes with the best clustering results (**Table S2**). We define these two independent subtypes as ICS-A and ICS-B. In subtype ICS-A, the scores of regulatory T cells and M0 and M2 macrophages were significantly higher than that of the subtype IC-B (**Figure 3**). Further, in subtype ICS-B, the proportion of B cells, CD8 T cells, memory activated CD4 T cells, memory resting CD4 T cells, NK cells, and M1 macrophages was significantly higher than that of the subtype ICS-A. On the other hand, no significant difference was observed in the proportion of native CD4 T cells, follicular helper T cells, eosinophils, and neutrophils between the two subtypes.

### 3.3 Kaplan−-Meier survival analysis

**Figure 4:**
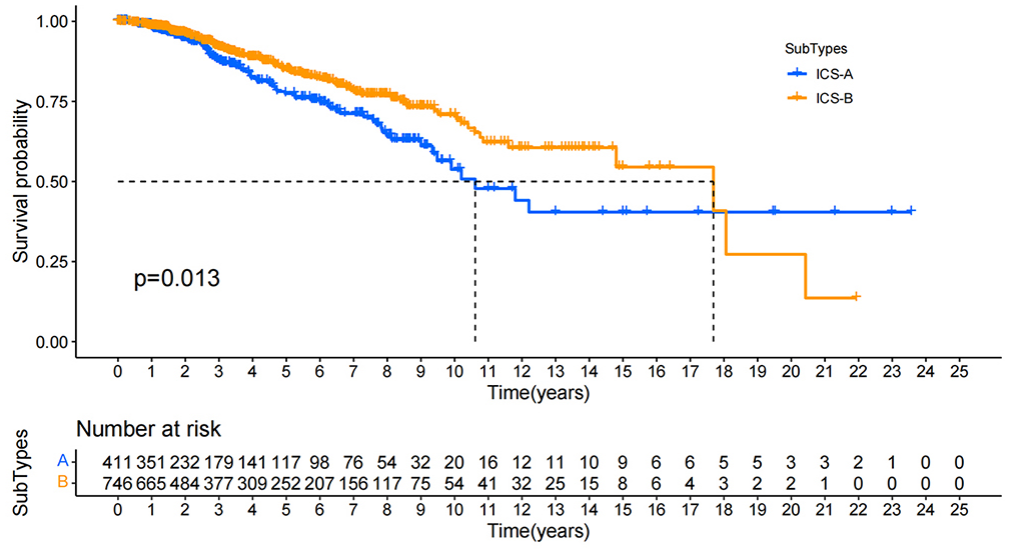
Kaplan-Meier survival curves for the overall survival of all breast cancer patients in the research cohort. Log rank test showed that the overall survival of ICS-A and ICS-B subtypes were significantly different (p-value = 0.013, p-values were adjusted using FDR correction).

In order to investigate the clinical significance of the subtype identification, we performed the Kaplan-Meier survival analysis on the OS of the two subtypes. The survival curve showed that the two subtypes had a significant difference in the OS, and the median survival time of the subtype ICS-B was 8 years longer than that of the subtype ICS-A (**Figure 4**).

### 3.4 Differential expression and drug sensitivity analysis of immune checkpoint molecules in the breast cancer

**Figure 5:**
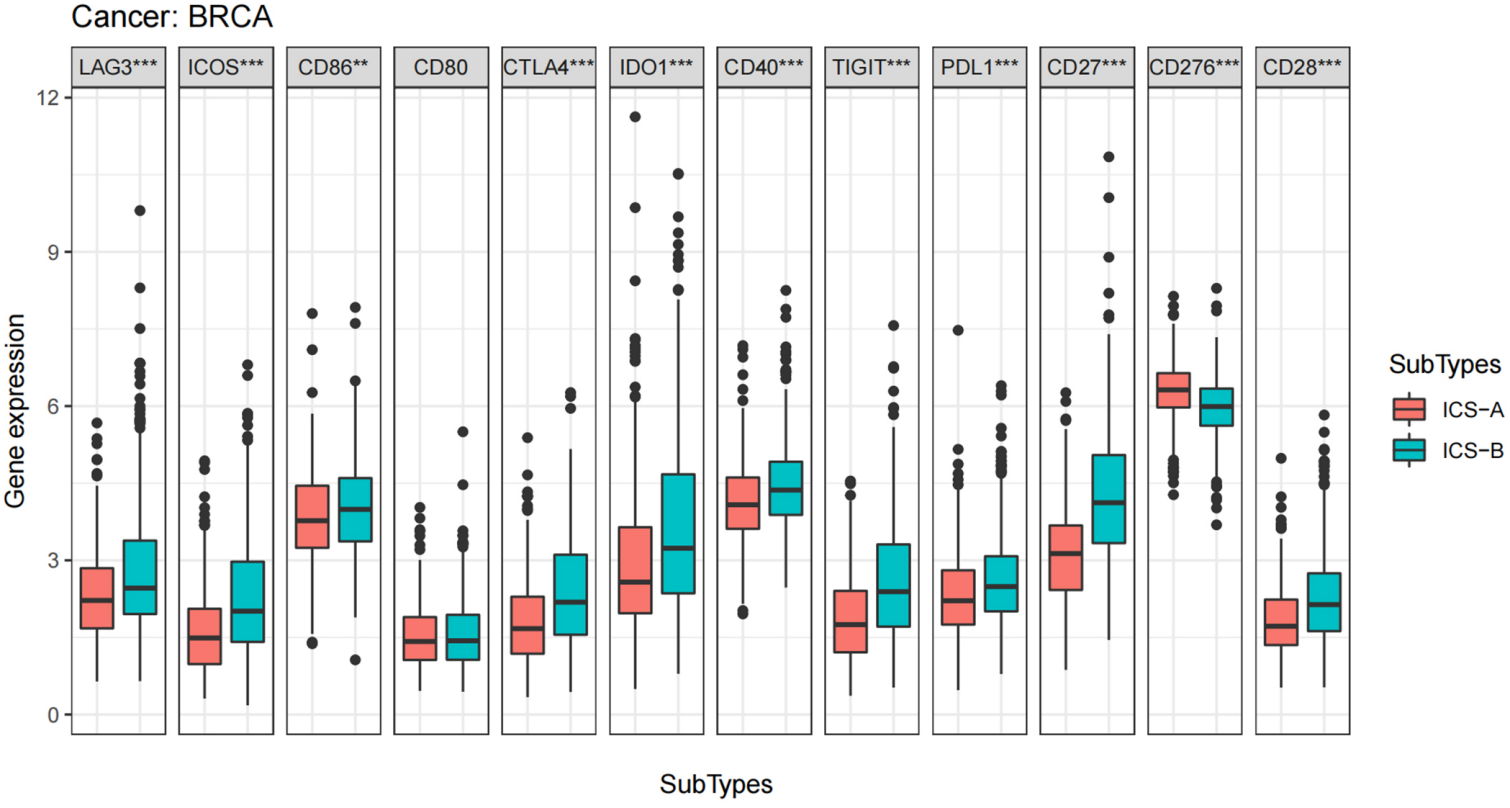
Boxplots of differential expression of immune checkpoint molecules. The analysis was performed using the Wilcoxon signed-rank test, *p < 0.05, **p < 0.01, ***p < 0.001 (p-values were adjusted using FDR correction).

**Figure 6:**
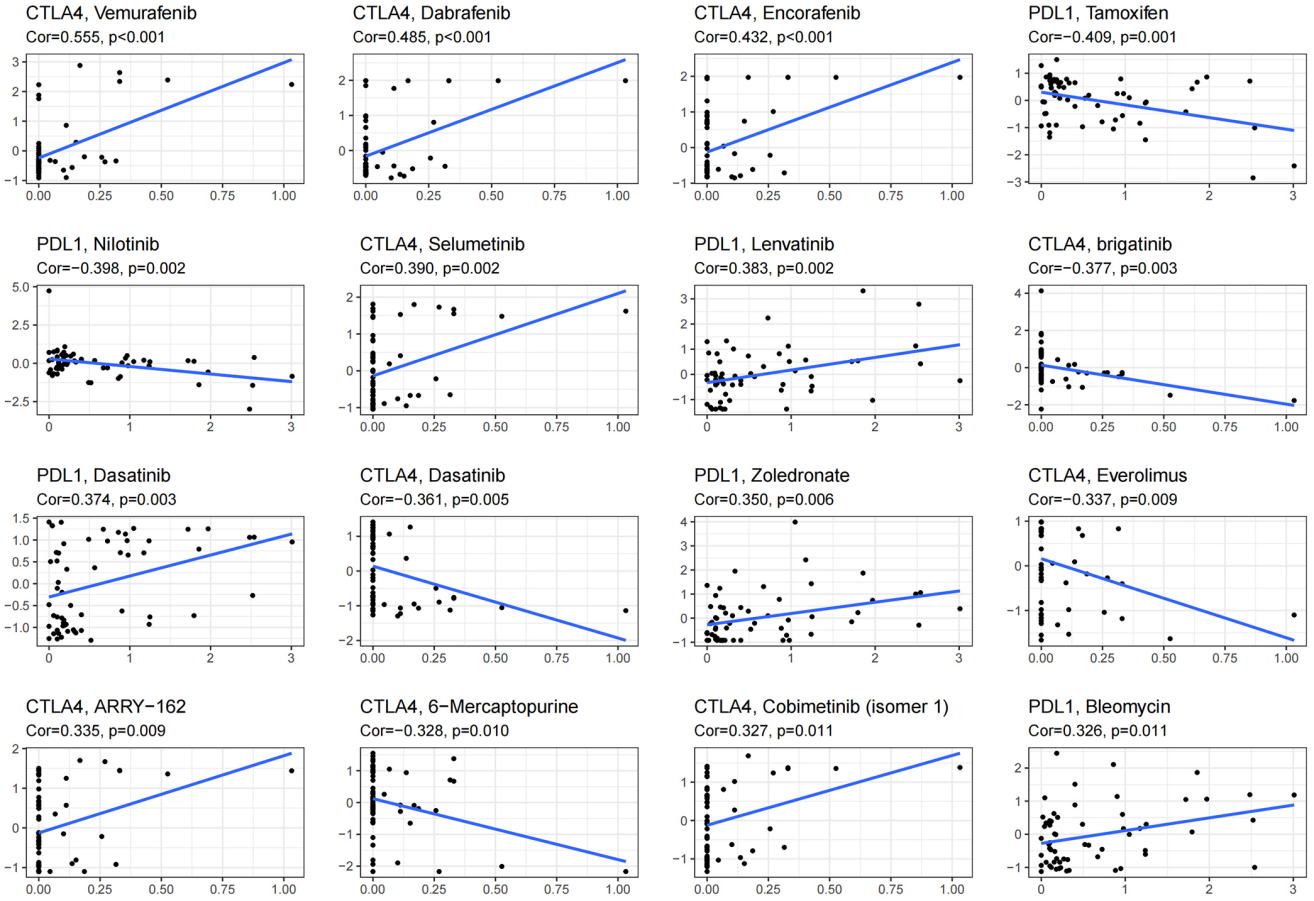
Scatter plot showing the association of expression of immune checkpoint molecules with drug sensitivity of NCI-60 cell line. Top 16 molecules are listed; PDL1 and CTLA4 were significantly associated with the drug sensitivity.

ICB therapy is currently the most promising immunotherapy for the treatment of breast cancer. We revealed differences in the expression levels of several key immune-modulatory molecules, including the co-stimulatory (CD27, ICOS, CD28, CD80, CD86, CD40, and CD276) and co-suppressive molecules (PDL1, CTLA4, LAG3, TIGIT, and IDO1) in the two subtypes. The expression levels of immuno-modulators (PDL1, CTLA4, LAG3, CD27, ICOS, CD28, CD86, CD40, TIGIT, and IDO1) in subtype ICS-B were significantly higher, while the CD276 levels were significantly lower than that of the subtype ICS-A (**Figure 5**). Furthermore, we investigated the expression of the immunomodulators in the 60 human cancer cell lines (NCI-60), and systematically tested the correlation between their expression levels in the NCI-60 cell lines with drug sensitivity of 218 FDA-approved chemotherapy drugs (**Table S3**). **Figure 6** shows the association between expression of immunomodulatory molecules (PDL1 and CTLA4) and drug sensitivity. We noticed that increased PDL1 expression was associated with increased cellular resistance to chemotherapy drugs such as Tamoxifen and Nilotinib; we also observed inverse associations of multiple genes to these drugs, Furthermore, PDL1 was associated with increased sensitivity of cells to Dasatinib (treatment for mantle cell lymphoma and chronic lymphocytic leukemia), while CTLA4 was associated with increased resistance of cells to Dasatinib.

### 3.5 Analysis of TMB in two subtypes

**Figure 7:**
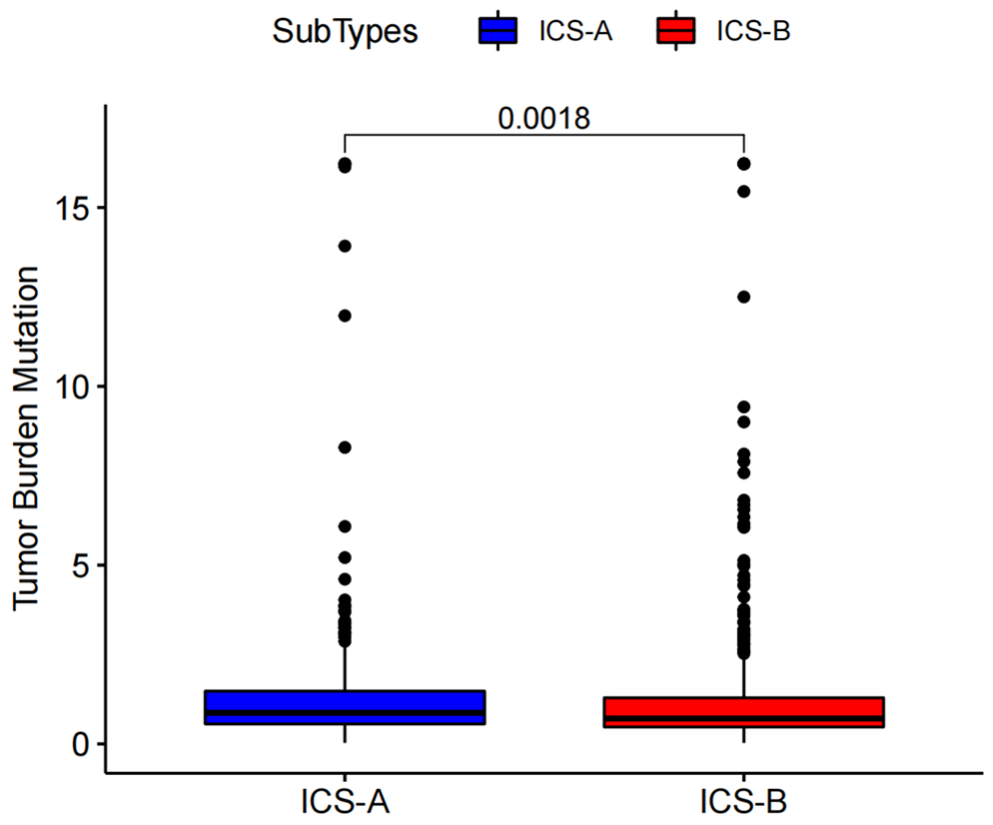
Tumor mutational burden (TMB) differences between the ICS-A and ICS-B subtypes. p-values were adjusted using FDR correction.

**Figure 8:**
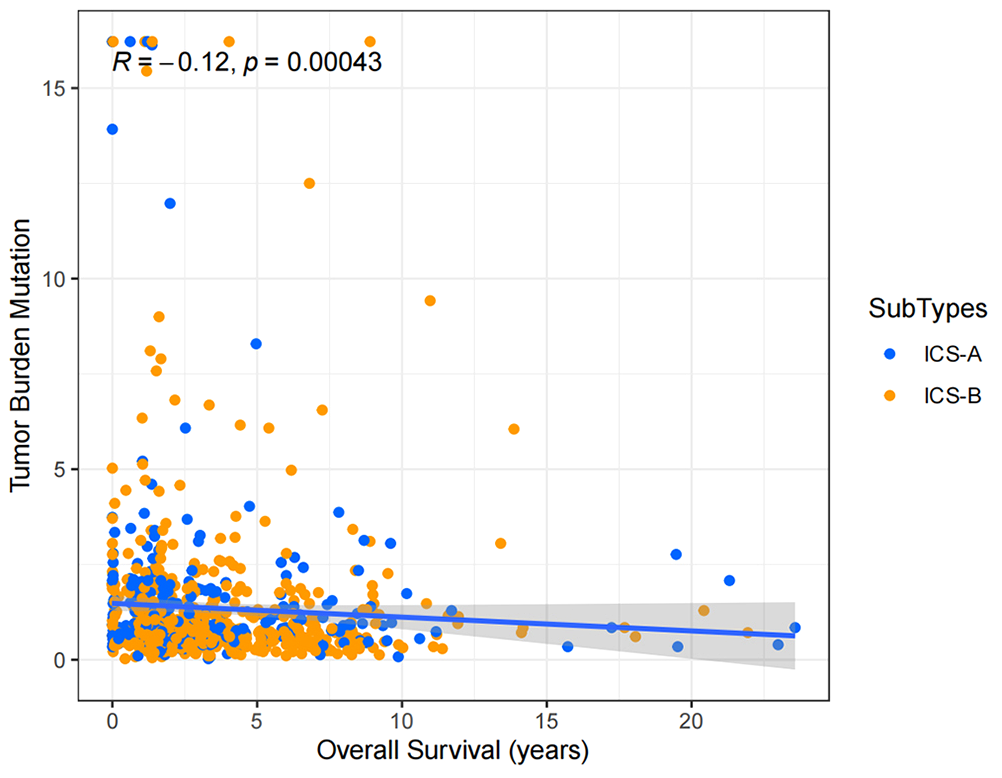
Tumor mutational burden (TMB) correlation analysis with the overall survival.

**Figure 9:**
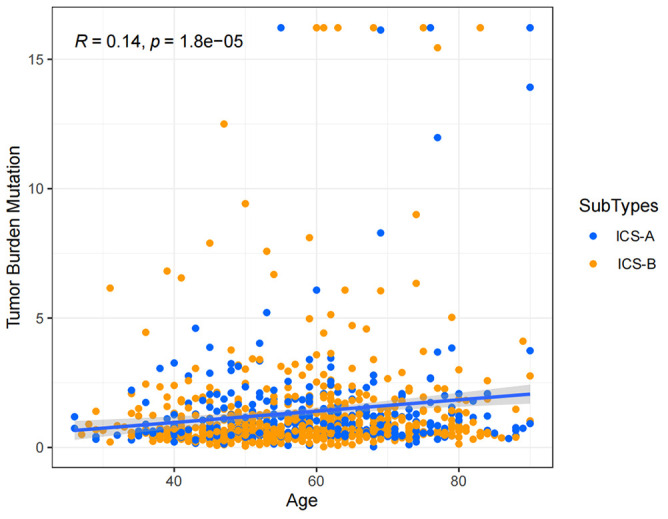
Tumor mutational burden (TMB) correlation analysis with age.

Considering the impact of TMB in tumor development, we further explored and revealed the correlation of TMB with OS and Age, respectively. We first counted the SNV of each sample in the TCGA cohort, and the frequency (number of samples) of the mutated genes in the research cohort (**Table S4**). The results showed that the number of samples with PIK3CA mutation was the largest, followed by TP53, TTN, CDH1, and GATA3. Further, the TMB subtype ICS-A was significantly higher than that of subtype ICS-B (Wilcoxon test p <0.001) (**Figure 7**). Furthermore, TMB showed significant negative and positive associations with the OS (Spearman coefficient: R = -0.12, p = 0.00043) and age (Spearman coefficient: R =0.14, p = 1.8e-05) (**Figures 8**, **9**).

### 3.6 Differentially expressed genes in the two subtypes

By using the Bayesian estimation test, more than 5000 DEGs were found between the subtypes ICS-A and ICS-B (**Table S5**). Further, under the conditions of p value <0.05,| *logFC*| >1, and 95% confidence interval (CI), a signature of 38 DEGs was used to identify the two subtypes. **Table 1** shows the list of genes identified.

**Table 1:** Comparison of different obfuscations in terms of their transformation capabilities.

Gene	logFC	AveExpr	t	P.Value	adj.P.Val	B
MMP9	-2.16300	6.53699	-22.74219	2.30436E-95	4.0453E-91	206.0615
SPP1	-1.57532	7.40448	-16.51827	2.41593E-55	2.1206E-51	114.9107
GZMK	1.37200	3.62741	16.30778	4.08304E-54	1.7919E-50	112.115
CD8A	1.13855	3.83799	15.98659	2.9213E-52	8.7254E-49	107.8928
ITM2A	1.16847	5.33790	15.98503	2.9822E-52	8.7254E-49	107.8724
CD27	1.11183	3.84288	15.47268	2.41841E-49	6.065E-46	101.2505
GZMA	1.23115	4.50821	15.34996	1.17782E-48	2.5846E-45	99.68555
CD3D	1.20354	4.59038	14.98294	1.27527E-46	2.4875E-43	95.05518
CD2	1.18523	4.97137	14.40682	1.70525E-43	2.1383E-40	87.94169
CD3E	1.04785	3.94658	14.37973	2.38055E-43	2.786E-40	87.61205
CCL19	1.79801	5.54616	13.89503	8.65444E-41	7.2347E-38	81.78719
SELP	1.03400	3.15338	13.45229	1.66462E-38	8.3492E-36	76.59265
ACKR1	1.54096	4.60494	13.44081	1.90484E-38	9.2887E-36	76.45952
SELL	1.09100	4.24668	13.42028	2.42319E-38	1.1497E-35	76.22184
CD79A	1.30591	3.78360	12.93794	6.41371E-36	2.3457E-33	70.71375
TNFRSF17	1.04404	2.42067	12.93594	6.56205E-36	2.351E-33	70.69118
NKG7	1.04241	4.27307	12.85253	1.69539E-35	5.9525E-33	69.75416
IL33	1.12487	3.51383	12.67761	1.22287E-34	3.4084E-32	67.80383
IGHM	1.72575	6.90470	12.48471	1.0554E-33	2.6852E-31	65.67671
C7	1.29024	3.28276	12.26408	1.20419E-32	2.7454E-30	63.27449
CCL5	1.00413	6.27016	12.24827	1.43191E-32	3.2227E-30	63.1036
IGKC	1.59129	8.71681	12.10846	6.57321E-32	1.3418E-29	61.60006
MS4A1	1.08781	2.23466	11.89404	6.62999E-31	1.1999E-28	59.32029
IGLL5	1.39436	4.96581	11.54786	2.58583E-29	3.9473E-27	55.7075
IGLV1-44	1.46962	5.91042	10.94526	1.24059E-26	1.5446E-24	49.62315
MFAP4	1.03166	5.87060	10.67499	1.81439E-25	1.9784E-23	46.98055
CXCL9	1.28566	6.23401	10.33092	5.10202E-24	4.9484E-22	43.69549
CHRDL1	1.11266	3.84926	10.09488	4.77912E-23	4.216E-21	41.49378
CCL21	1.21797	4.35651	9.90108	2.90555E-22	2.4761E-20	39.71812
IGHD	1.06524	2.92884	9.80581	6.98186E-22	5.6744E-20	38.85589
MMP13	-1.08928	4.17293	-9.37678	3.31451E-20	2.3557E-18	35.06133
MMP12	-1.03738	2.61418	-9.27906	7.82465E-20	5.2831E-18	34.21744
IGLV6-57	1.17385	4.73478	9.11611	3.22241E-19	2.0203E-17	32.82725
COL11A1	-1.15667	5.69851	-9.08603	4.17467E-19	2.5625E-17	32.57302
ADH1B	1.16813	3.34122	9.08188	4.32622E-19	2.6462E-17	32.53801
MMP1	-1.25655	4.14037	-8.85569	2.96053E-18	1.6292E-16	30.6501
IGHG1	1.06179	7.57753	7.55540	8.29792E-14	2.9428E-12	20.61969
CXCL13	1.04036	4.59093	7.26950	6.51845E-13	2.0958E-11	18.60688

Condition employed: pvalue <0.05, | logFC | >1, and 95% confidence interval.

The results were obtained by using the R “limma” package Bayesian test.

### 3.7 Gene ontology and KEGG pathway enrichment analysis

**Figure 10:**
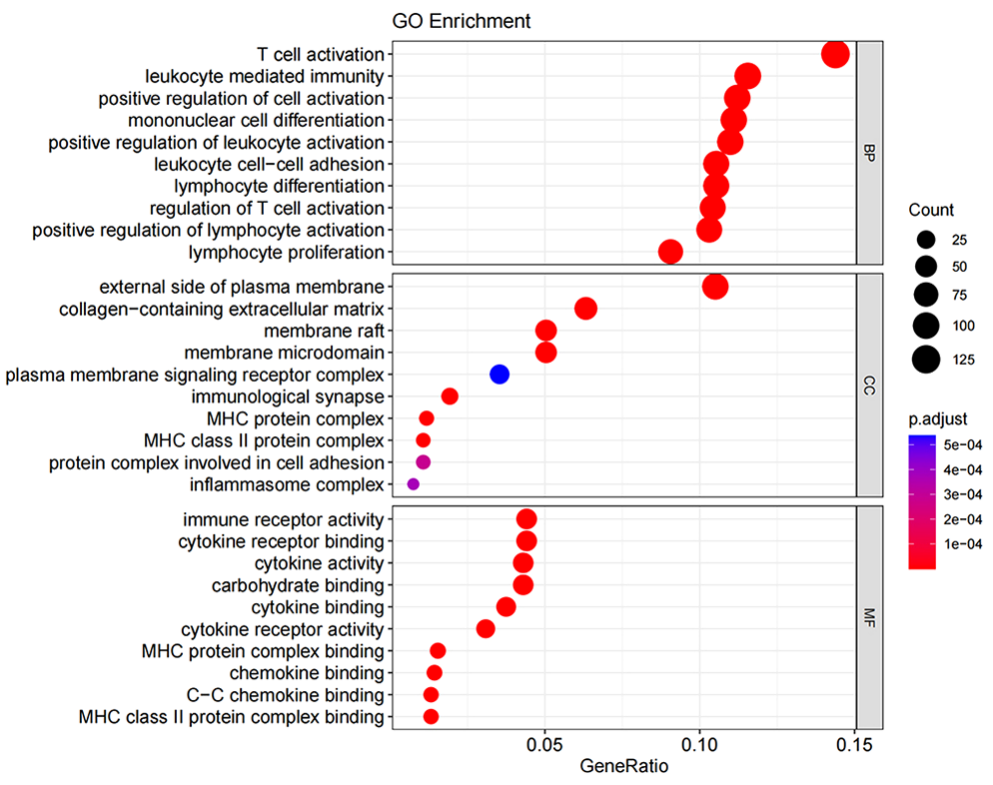
Gene ontology term enrichment for DEGs of ICS−A and ICS−B subtypes. The result shows the top 10 significantly enriched terms on the BP, CC, and MF.

**Figure 11:**
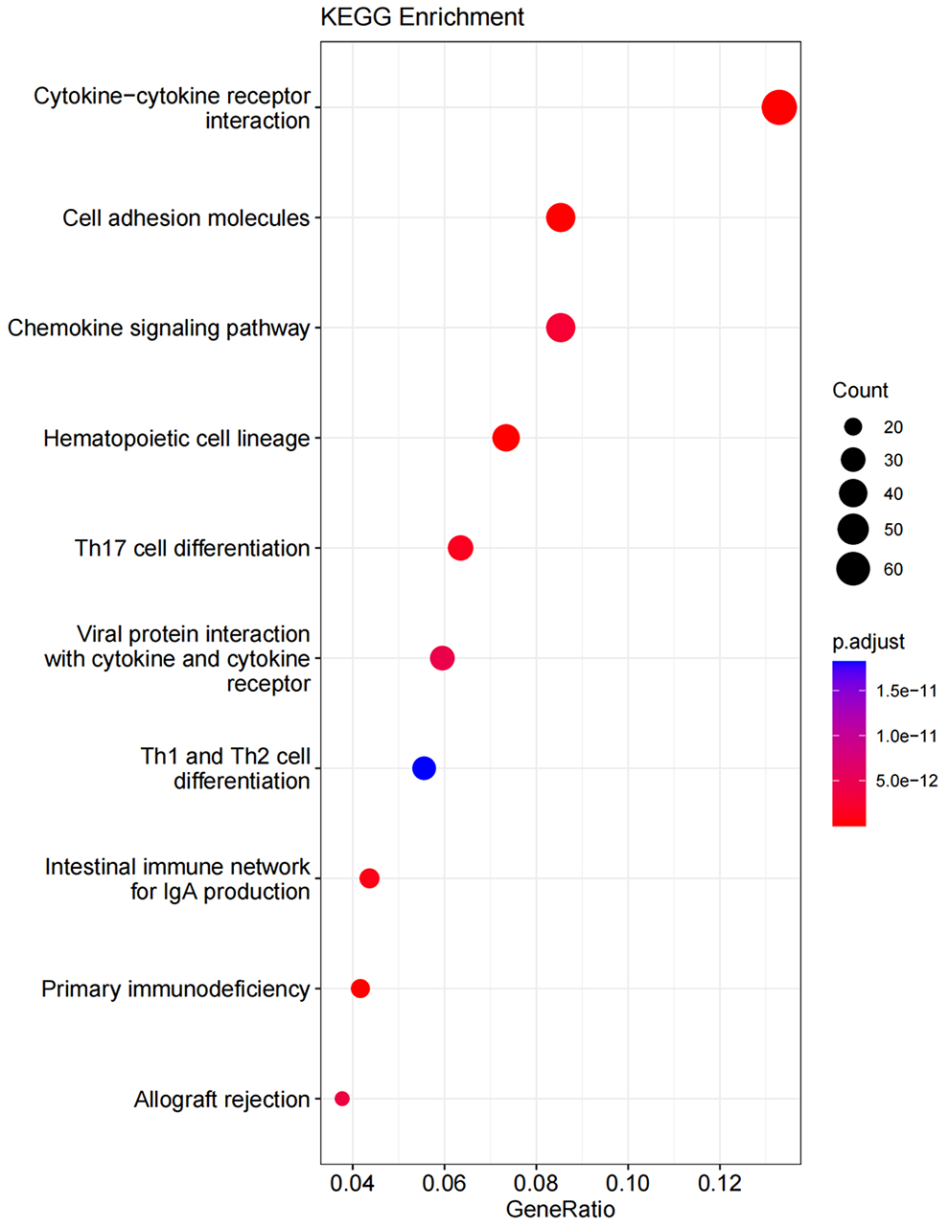
KEGG pathway enrichment for DEGs of ICS-A and ICS-B subtypes. The result shows the top 10 significantly enriched pathways.

GO enrichment analysis covers three domains: cellular component (CC), molecular function (MF), and biological process (BP). **Figures 10**, **11** show the results of GO terms and KEGG pathways enrichment analysis, respectively. The top 5 BPs were significantly enriched in the T cell activation, leukocyte mediated immunity, positive regulation of cell activation, mononuclear cell differentiation, and positive regulation of leukocyte activation. The CC analysis revealed that DEGs were mainly enriched in the external side of the plasma membrane, membrane raft, and membrane micro domain, while the MF significantly enriched in immune receptor activity, cytokine receptor binding, cytokine activity, and carbohydrate binding. KEGG pathway analysis showed that cytokine-cytokine receptor interaction was the most significant pathway for the DEGs enrichment, followed by cell adhesion molecules, chemokine signaling pathway, and hematopoietic cell lineage.

### 3.8 Breast cancer subtype classifier based on the neural network

**Figure 12:**
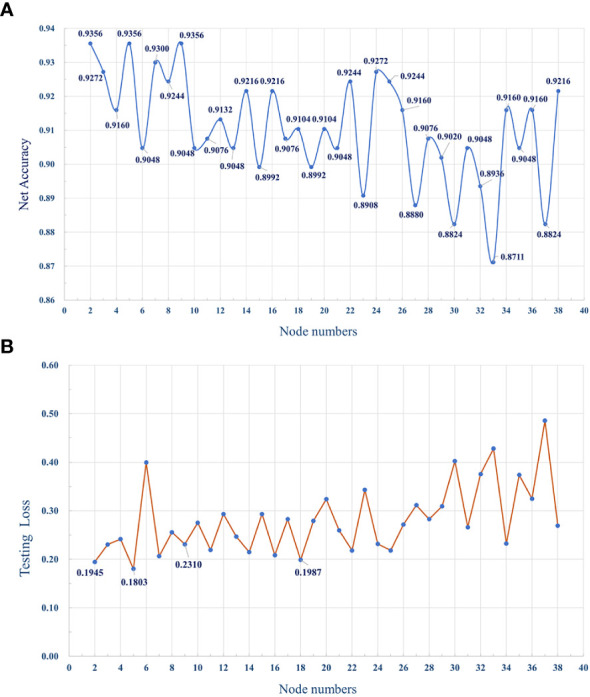
**(A)** The dotted line graph shows the accuracy of the MLP classifier, with different number of nodes in the hidden layer. **(B)** The average loss of the model with different numbers of hidden layer nodes during testing.

The MLP classifier for identifying subtypes was defined as three layers, including the input, hidden, and output layers, while the number of nodes in each layer was respectively defined as 38, 5, and 2, according to the training and testing results of the program. **Figure 12A** shows the accuracy of the classifier model with different numbers of nodes in the hidden layer.

## 4 Discussion

**Figure 13:**
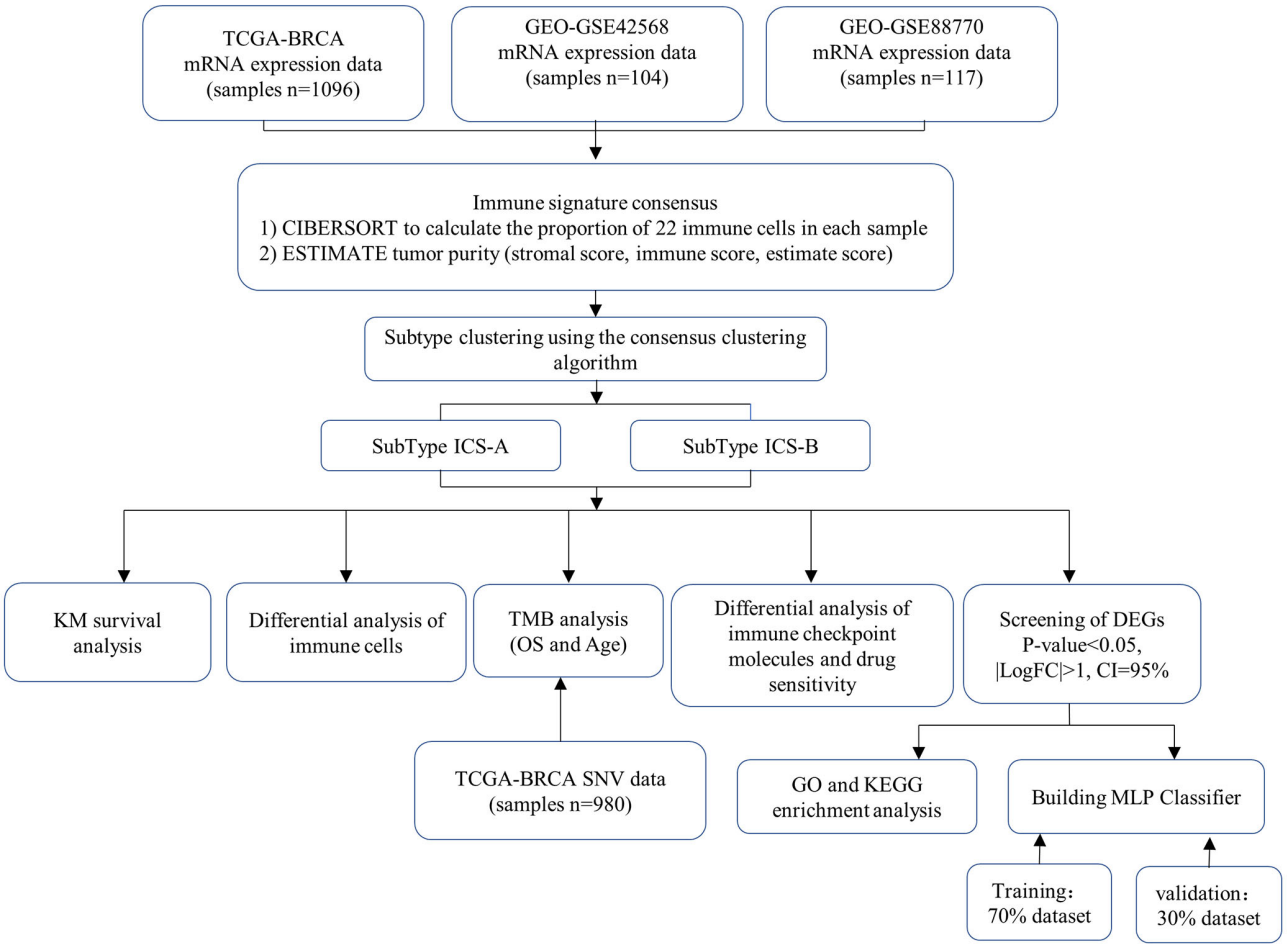
Overview of the study design.

We performed a detailed and comprehensive assessment of the TIICs in breast cancer using the research cohort from TCGA and GEO databases (**Figure 13**). Compared with previous studies (27, 29–32), this study determined the composition of TIICs in breast cancer, and the research cohort was divided into two subtypes, ICS-A and ICS-B, according to the composition of TIICs. A 38-ene signature tumor marker was identified and a classifier for subtype identification was developed using a deep learning framework. Our study confirmed that the proportion of immune cells and the expression level of immune checkpoint molecules in subtype ICS- were significantly higher than those in subtype ICS-A. Further, subtype ICS-B had better OS, suggesting that it is more suitable for immune checkpoint blockade therapy than subtype ICS-A. At the same time, we also conducted the drug sensitivity (FDA-approved chemotherapy drugs) analysis of theimmune checkpoint molecules that provided a reference for the selection of these drugs for breast cancer patients.

The TIME has an important impact on tumor diagnosis, treatment, and prognosis. The immune score has been used in renal and lung cancer studies in terms of estimating the relative proportion fraction of TIICs in tumors, and has shown its prognostic value (36–38). In this study, subtype ICS-B (with higher levels of CD8 and memory resting CD4 T cells) was found to be associated with better OS. CD8 T cells are key anti-tumor effector T cells, and CD4 T cells can be further differentiated to perform various functions (for instance, to differentiate into CD8 memory T cells to suppress tumor growth) (36, 39, 40); however the M2 macrophages can also suppress anti-tumor immune responses by secreting multiple mediators such as the inhibitory cytokines IL-10 or TGF-B, down-regulating antitumor immune response, promotion of angiogenesis, enhancement of cancer cell proliferation, invasion, intravascular penetration, and spread have been metastasized (41–44). This suggests that the immune subtype ICS-A with a lower proportion of CD8 T cells but a higher proportion of M2 macrophages may have an immunosuppressive (immune rejection) phenotype, and M2 macrophages or CD8 T cells may provide a therapeutic target for future breast cancer immunotherapy.

TMB has been recognized as a predictive marker of immunotherapy response and a prognostic marker in various tumor types (45–48). In this study, the TMB level of immune subtype ICS-A was significantly higher than that of the subtype ICS-B, indicating that patients with subtype ICS-A may produce more neo-antigens and will adversely affect the patient survival. Therefore, subtype ICS-B is indicative of better OS. This idea is supported by the correlation analysis between the TMB and OS. The level of TMB was significantly and negatively correlated with the OS in breast cancer patients. Further, since the TMB and age showed positive interaction, older patients had relatively higher TMB. This is consistent with a recent study showing that TMB increases with age, while the T cell receptor decreases (49). This provides unique insights into clinical prognostic diagnosis.

Statistical analysis of SNV in the research cohort showed that PIK3CA had the highest mutation frequency. PIK3CA is a catalytic subunit of the key proto-oncogene PI3K in the PI3K-Akt signaling pathway. Mutation of PIK3CA can lead to enhanced kinase activity, which in turn continuously stimulates downstream AKT (50), increases cell invasion and metastasis, and promotes tumor development. PIK3CA is located on chromosome 3, with a total of 20 exons, and 80% of PIK3CA mutations occur in the two hotspot regions of the helical region and the kinase region. The three most common mutations are H1047R on exon 20, and E542k and E545K on exon 9. Many studies have confirmed the existence of PIK3CA mutations in various human solid tumors, and its positive rate in breast cancer can reach 30-40% (51–53). This result has also been confirmed in our study. There were a total of 980 samples in our research cohort, of which 322 samples had PIK3CA mutation, with a positivity rate of 32.86%. This indicates that PIK3CA can be used as a prognostic molecular biomarker and therapeutic target for breast cancer. The development and use of drugs targeting PIK3CA to block the PI3Ks pathway will play an effective role in the treatment of breast cancer. In recent years, the deep learning framework in the field of artificial intelligence is gradually being applied in various disciplines and industries. Although the interpretability of deep learning frameworks is still debated, their ability to solve bioinformatics problems requires further investigation. It has shown powerful functions in bioinformatics such as protein structure prediction (54, 55), protein−protein interaction prediction (56, 57), RNA structure prediction (58, 59), drug small molecule interaction prediction (60, 61), and drug design (62–64). Based on the identification of breast cancer immune subtypes, we designed and developed a subtype ICS−A classifier based on a deep learning framework.

**Figure 14:**
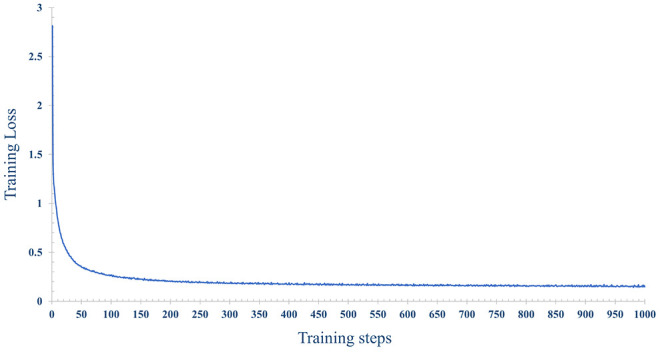
The change process of the loss, with the increase of training epochs, when the number of nodes is 5 in the hidden layer.

**Figure 15:**
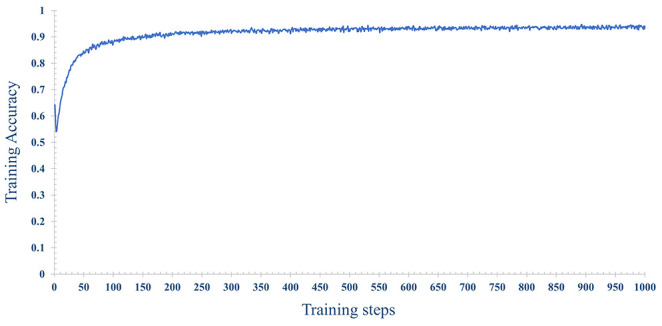
The change process of the accuracy rate, with the increase of training epochs, when the number of nodes is 5 in the hidden layer.

In order to improve the prediction accuracy of the model, we used 70% of the data for model training and 30% of the data for model testing. The number of iterations epoch is 1000 times. The loss and accuracy of the model during the training process are shown in **Figures 14**, **15** shown. As the number of training increases, the loss of the model decreases and tends to stabilize. The accuracy improves continuously with the increase of training times, and tends to stabilize after more than 200 times. Further, comparative experiments were conducted using other machine learning models, and **Table 2** shows the highest accuracy of each model on the test dataset. The results show that Naive Bayes has the lowest accuracy (89.36%), and the accuracy rates of SVM, RF, MLP are 92.99%, 91.59% and 93.56%, respectively. The prediction accuracy of the MLP model on the test dataset is slightly higher than that of the SVM by 0.57%. However, with tuning of the MLP hyperparameters (eg, number of model layers, number of iterations for training), the prediction performance could be improved. **Figure 12A** shows the accuracy of models trained with different numbers of nodes in the hidden layer. When the number of nodes were 2, 5 and 9 respectively, the accuracy was the highest (93.56%), while with the number of nodes at 33, the accuracy was the worst (87.11%); However, when the number of hidden layer nodes is 5, the model obtains the smallest loss during testing(**Figure 12B**). Hence, the number of nodes in the hidden layer of the model was finally determined to be 5. This classifier can effectively assist clinicians in the diagnosis and subtype identification of breast cancer.

**Table 2:** Accuracy comparison of machine learning models.

Model Types	Testing Accuracy
SVM Model	92.99%
Naive Bayes Model	89.36%
Random Forest Model	91.59%
MLP Model	93.56%

In conclusion, the identification of breast cancer subtypes based on the immune signature in the tumor microenvironment can assist clinicians to effectively and accurately assess the progression of breast cancer and formulate different treatment strategies for different subtypes. In the present study, we detailed the immune infiltration landscape of the study cohort and demonstrated the clinical utility of immune−based subtyping. Further, this study explored the differences in immune checkpoint molecules, DEGs, and pathway enrichment between the two subtypes, and revealed that TMB in breast cancer patients was associated with OS and age. These findings have the potential to provide a new approach for the targeted therapy of breast cancer and lay a theoretical basis for the use of chemotherapy drugs for patients. Finally, we developed a subtype classifier with high robustness and accuracy, which can effectively assist clinicians in medical diagnosis.

## Data availability statement

The original contributions presented in the study are included in the article/**Supplementary Material**. Further inquiries can be directed to the corresponding author.

## Author contributions

XY and YZ contributed to conception and design of the study. XY and XS contributed to the collection and collation of data. XY and WJ performed the statistical analysis. XY and XX wrote the first draft of the manuscript. XS, WJ, and HP wrote sections of the manuscript. All authors contributed to manuscript revision, read, and approved the submitted version.
