# Supplementary material for: Immune subtype identification and multi-layer perceptron classifier construction for breast cancer
Source: Front Oncol. 2022 Dec 8;12:943874. doi: 10.3389/fonc.2022.943874 (PMC9780074; doi:10.3389/fonc.2022.943874)
Supplement: Supplementary file 1 [file DataSheet_1.zip › Supplementary Material/Supporting Information.docx]

**List of Supporting Information:**

Table S1. The estimated results using CIBERSORT and ESTIMATE algorithms.

Table S2. Immune subtype clustering results by using the consensus clustering algorithm.

Table S3. The correlation between the immune checkpoint molecules expression levels in the NCI-60 cell lines with drug sensitivity of 218 FDA-approved chemotherapy drugs.

Table S4. Mutation frequency of each mutated gene in the research cohort.

Table S5. Differentially expressed genes between the two subtypes, p value <0.01.
